# A Simple Method to Evaluate the Length of Poly(A) Tails in mRNA

**DOI:** 10.3390/mps9040109

**Published:** 2026-07-13

**Authors:** Jonas Mumenthaler, Maximilian Feldmann, Shahab Mamaghani, Rocco Roberto Penna, Julia Frei, Natalia Jarzebska, Mark Mellett, Emmanuella Guenova, Thomas Kündig, Severin Lauchli, Steve Pascolo

**Affiliations:** 1 Department of Dermatology, University Hospital Zürich (USZ), University of Zürich (UZH), 8091 Zürich, Switzerland; jonas.mumenthaler@usz.ch (J.M.); maximilian.feldmann@usz.ch (M.F.); shahab.mamaghani@biomix.ch (S.M.); rocco.penna@usz.ch (R.R.P.); julia.frei@usz.ch (J.F.); mark.mellett@usz.ch (M.M.); thomas.kuendig@usz.ch (T.K.); severin.laeuchli@stadtspital.ch (S.L.); 2 Faculty of Medicine, University of Zürich, 8091 Zürich, Switzerland; 3 Faculty of Science, University of Zürich, 8091 Zürich, Switzerland; 4 Lausanne University Hospital (CHUV) and University of Lausanne, 3001 Lausanne, Switzerland; emmanuella.guenova@jku.at

**Keywords:** poly(A) tail, mRNA, in vitro transcription, ivt mRNA, ECHO probe

## Abstract

Synthetic mRNA produced by in vitro transcription is the active pharmaceutical ingredient of approved vaccines and of many drugs under development. It typically contains a 3′ poly(A) tail required for optimal stabilization and translation of the mRNA. The average length of the poly(A) tail in the produced mRNA cannot be precisely predicted and consequently must be measured using complicated technologies such as reverse transcription and sequencing or cleavage of the poly(A) tail and analysis by capillary electrophoresis or mass spectrometry. We report an accelerated method to evaluate the average poly(A) tail length in synthetic mRNA, which benefits from the fact that thiazole orange is fluorescent only when in close proximity of nucleic acid. An oligo(dT)_12_ oligonucleotide having thiazole orange at its extremities emits a fluorescence signal proportional to the amount of poly(A) sequence available. Using a titration curve made with known amounts of poly(A) RNA oligonucleotide, the thiazole orange oligo(dT)_12_ oligonucleotide can instantly indicate the average length of the poly(A) tail in an mRNA sample. This fast and easy method can be used in any laboratory to determine the size of the poly(A) tail in in vitro-transcribed mRNA produced for research, pre-clinical studies and clinical trials.

## 1. Introduction

The synthesis of mRNA, such as that used in approved vaccines against SARS-CoV2 [1,2,3] or Respiratory Syncytial Virus (RSV) [4], as well as in many new drugs currently under development [5,6], is achieved by in vitro transcription (ivt) of a DNA template [7], initially using purified RNA polymerase [8] and, after 1984, recombinant RNA polymerase [9]. The first report in 1984 on the functionality of synthetic mRNA described the use of mRNA encoding the *Xenopus* β-globin protein [10]. On top of the coding sequence, this ivt mRNA contained the endogenous 5′ and 3′ untranslated regions (UTRs) of the *Xenopus* β-globin gene and a poly(A)_23_(C)_30_ tail. Since then, ivt mRNAs are based on this format and frequently contain globin 5′ and 3′ UTRs and the poly(A) tail encoded in the DNA. The first report demonstrating the possibility of expressing proteins in animals through injection of synthetic mRNA [11], as well as the initial studies showing vaccination in mice using direct injection of synthetic mRNA [12] and the first clinical studies, initiated in 2003, to evaluate ivt mRNA vaccines in humans [13], employed this type of mRNA. Currently, in order to obtain the 3′ poly(A) tail that is needed for the functionality of ivt mRNA, there are two distinct possibilities:

- The poly(A) sequence is contained in the DNA template. In this case the RNA polymerase will copy it into the neo-synthesized mRNA; however, it will not always incorporate all As: the RNA polymerase may stop on this homopolymeric sequence, particularly when the ATP pool is being reduced during transcription due to heightened consumption of the nucleotide at the beginning of the reaction. Therefore, the obtained mRNAs will all have poly(A) tails of varying lengths, often lower than the length of the poly(A) sequence in the DNA matrix.
- The poly(A) sequence is added on the ivt mRNA post-transcriptionally using a poly(A) polymerase and ATP. In this case the number of adenosine residues added will depend on several factors: the concentration of ATP in the reaction, the efficacy of the poly(A) polymerase, the accessibility of the 3′ end (i.e., it should not be sequestered within secondary or tertiary RNA structures), the amount of available 3′ ends (longer mRNAs provide less 3′ ends per microgram of mRNA), and the duration of the reaction. Commercial manufacturers provide protocols to introduce hundreds of As on average (https://www.neb.com/en/protocols/2014/08/13/poly-a-tailing-of-rna-using-e-coli-poly-a-polymerase-neb-m0276 (accessed on 9 July 2026)). Again, here, some mRNAs will have no As (due to failure of being elongated by the poly(A) polymerase) and some will have more than 200 As (as a result of efficient elongation).

Therefore, regardless of the method used, the size of the poly(A) tail in synthetic mRNA is not precisely predictable. In pharmaceutical production of ivt mRNA, there is a quality control step that must be performed to accurately measure the poly(A) tail length (https://www.ema.europa.eu/en/documents/scientific-guideline/draft-guideline-quality-aspects-mrna-vaccines_en.pdf (accessed on 9 July 2026)). Such a quality control measure to determine the poly(A) tail length can be accomplished by reverse transcription and sequencing, or cleavage of the poly(A) tail followed by mass spectrometry or capillary electrophoresis analysis. These methods are complex and can be difficult to implement in research laboratories where researchers generate their own ivt mRNA. They are also labour-intensive and costly in pharmaceutical conditions for individualized anti-cancer therapies (where mRNAs are produced tailored for each patient).

We developed a simple and rapid method that evaluates the length of the poly(A) tail in ivt mRNA samples using thiazole orange coupled to a poly(dT) oligonucleotide. Thiazole orange is a fluorescent dye that exhibits minimal fluorescence in its free state but shows a significant increase in fluorescence upon binding to nucleic acid (DNA or RNA) [14,15,16]. This property makes it valuable for various applications in molecular biology, including the development of ECHO (Exciton-Controlled Hybridization-Sensitive Fluorescent Oligonucleotide) probes [17]. These probes utilize the unique fluorescence properties of thiazole orange or its derivatives for the detection of target nucleic acids. Biological applications of ECHO probes include: Fluorescence In Situ Hybridization (FISH) for visualizing specific nucleic acid sequences within cells or tissues, monitoring mRNA, lncRNA, and miRNA dynamics in living cells, Single Nucleotide Polymorphism (SNP), and pathogen detection (for example, in SARS-CoV-2 testing kits).

Here, we show that poly(dT)_12_ DNA oligonucleotides incorporating thiazole orange at the 5′ end, 3′ end or on both ends exhibit fluorescence upon hybridisation with complementary poly(A) RNA sequences. Using a titration of defined amounts of synthetic poly(A)_60_ RNA oligonucleotide against a fixed amount of the poly(dT) DNA oligonucleotide, and measuring the resulting fluorescence signal, yielded reproducible standard curves. By applying this method to synthetic mRNA samples of unknown poly(A) tail length in separate wells and using the resulting fluorescence signal, we are able to quantify, based on the previously generated standard curves, the amount of poly(A) in each sample, allowing the estimation of the average poly(A) tail length in the mRNA.

## 2. Materials and Methods

### 2.1. Oligonucleotides

The oligo(dT) homopolymer consisting of 12 consecutive deoxythymidines was made on a chemical synthesizer and coupled with thiazole orange at its 5′ end or at its 3′ end or at both (Biosynth Inc, San Diego, CA, USA). The oligo(A) homopolymer consisting of 60 consecutive ribo-adenosine was also made on a chemical synthesizer (Microsynth, Zurich, Switzerland). All oligonucleotides were resuspended at 100 pmol per µL in pure water and stored frozen. DNA primers for PCR were made on a chemical synthesizer (Microsynth, Zurich, Switzerland).

### 2.2. Production of Synthetic Messenger RNA by In Vitro Transcription

DNA templates were plasmids having the sequence of gaussia luciferase, firefly luciferase or mCherry in the “min” design (5′ CAAG UTR, no 3′ UTR [18]), an upstream modified T7 promoter accommodating the incorporation of the CleanCap^®^ Reagent AG (TriLink BioTechnologies Inc., San Diego, CA, USA) and a downstream poly(A)_120_ sequence interrupted with a single G residue: A_60_GA_60_. Unique restrictions sites were incorporated immediately after the stop codon or after the poly(A) tail so that the plasmids could be used to make mRNA without a poly(A) tail (plasmid linearized immediately after the stop codon) or with a poly(A) tail (plasmid linearized immediately after the poly(A) sequence). Synthetic mRNAs were produced by in vitro transcription using the HiScribe™ T7 mRNA kit (New England Biolabs, Ipswich, MA, USA). Therefore, the mRNAs made from plasmids linearized after the poly(A) tail were co-transcriptionally poly-adenylated at the 3′ end using DNA templates having a defined stretch of 120 As. The transcription of mRNA was performed in the presence of 8 mM of each ATP, CTP, GTP, and methyl-1 pseudouridine triphosphate. After transcription, DNA was degraded by DNase I (New England Biolabs). Afterward, synthetic mRNA was recovered by LiCl precipitation. RNA was diluted in RNase-free water, and the concentration was adjusted to 1 mg/mL using a Nanodrop (Thermo Scientific, Waltham, MA, USA). The quality and integrity of ivt mRNAs were checked using agarose gel electrophoresis. The mRNAs were stored at −20 °C.

## 3. Results

### 3.1. Optimization of ECHO Probe Fluorescence Conditions

**Figure 1:**
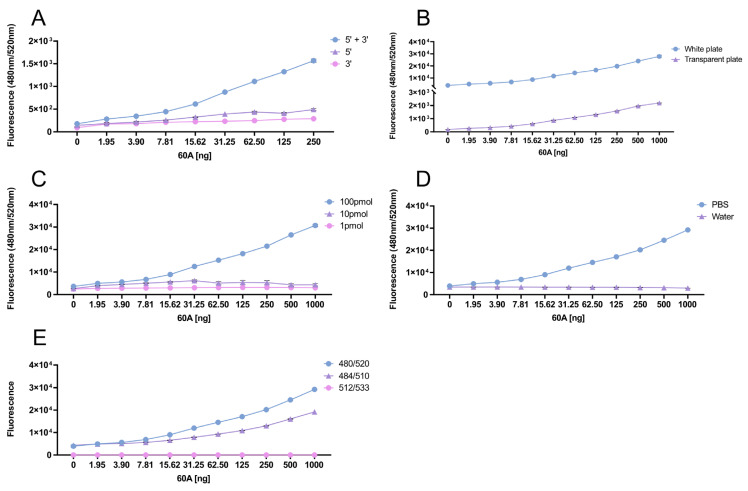
Optimization of the ECHO probe fluorescence assay. (**A**) Oligo(dT)_12_ deoxyoligonucleotide labelled with thioazol orange only at the 5′ end “5′”, only at the 3′ end “3′” on or both ends (5′ + 3′) were added (100 pmol per well, transparent plate) to increasing amounts of poly(A)_60_ chemically synthesized RNA (“60A”): 1.95, 3.9, 7.81, 15.62, 31.25, 62.5, 125, 250, 500, and 1000 ng of poly(A)_60_ corresponding to 0.10, 0.20, 0.40, 0.81, 1.61, 3.22, 6.44, 12.89, 25.77, and 51.55 pmol respectively. Then, 20 min later fluorescence was recorded with ex/em at 480/520 nm. (**B**) Oligo(dT)_12_ deoxyoligonucleotides labelled with thioazol orange at the 5′ and 3′ ends (ECHO-53) were added 100 pmol per well, in transparent or white plates to increasing amounts of poly(A)_60_ chemically synthesized RNA and measured at 480/520 nm. (**C**–**E**), the assay was performed in white plates as in (**B**) but (**C**) the amount of ECHO-53 oligonucleotide used was 100 pmol,10 pmol or 1 pmol per well, or (**D**) the poly(A)_60_ and ECHO-53 were put either in pure water (H_2_O) or PBS, or (**E**) the measurements were made at different excitation/emission wavelengths as indicated. All assays were performed in wells of a 96-well plate, in a final volume of 100 µL.

A deoxyoligonucleotide homopolymer consisting of 12 unmodified dT residues was synthesized and coupled at its 5′-, 3′- or both ends to thiazole orange (“ECHO probe”). These oligonucleotides were resuspended at 100 pmol/µL. When mixed with increasing amounts of a poly(A) RNA oligonucleotide of 60 nucleotides (poly(A)_60_ RNA) in a final volume of 100 µL of Phosphate-Buffered Saline (PBS) containing a fixed amount of 100 pmol of ECHO probe, we recorded increasing fluorescence signals using the 480 nm excitation (“ex”) and 520 nm emission (“em”) channels on a spectrophotometer (Figure 1A). Emission in the presence of poly(A) was higher when the ECHO probe had thiazole orange on both termini compared to only either the 3′ or 5′ end. The ratio between autofluorescence (samples without poly(A)) and fluorescence in the presence of 250 ng of poly(A)_60_ RNA was 491.5/140.5 = 3.5 for the 5′ thiazole orange probe, 288/90 = 3.2 for the 3′ thiazole orange probe and 1568/177.5 = 8.8 for the 5′ + 3′ thiazole orange probe. Thus, subsequent experiments were performed using only the 5′ + 3′ thiazole orange oligo-(dT)_12_ deoxyoligonucleotide termed “ECHO-53”. Experiments were conducted in white or transparent 96-well plates, with the measured fluorescence being 10-fold greater than the former (Figure 1B). The required amount of ECHO-53 was 100 pmol with lower amounts showing little fluorescence (Figure 1C). Successful hybridisation and the resulting fluorescent signal were only observed in PBS, with no signal obtained inwater, reflecting the need of salts to achieve hybridisation between complementary nucleic acids (Figure 1D). At least 40 mM NaCl was required to get full hybridisation (Supplementary Figure S1). Thiazole orange fluorescence in the vicinity of nucleic acid is reported to take place optimally at the ex/em 512/533 [14,16,17]. However, in our experiments, this setting did not give a detectable signal while excitation/emission settings of 484/501 or, even more effectively, 480/520 resulted in strong signals (Figure 1E). The fluorescence signal remained stable over hours after mixing of the RNA and ECHO-53 (Supplementary Figure S2). The optimal range appeared to be from 7 to 1000 ng of poly(A)_60_ RNA per well using our equipment, as over 1000 ng signals became saturated.

### 3.2. Evaluating the Size of the Poly(A) Tail Length in Generated Ivt mRNA Using the ECHO Probe

**Figure 2:**
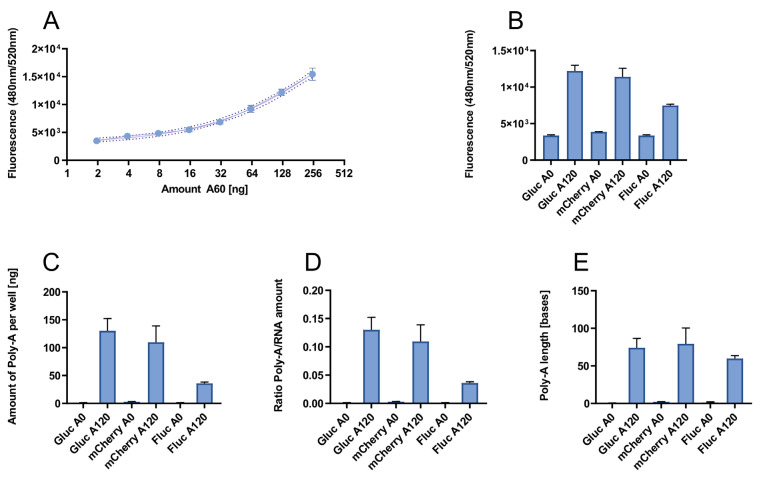
Evaluating the average poly(A) length in mRNA samples. (**A**–**E**) The assay was performed in white 96-well plates with titrating amounts of poly(A)_60_ RNA oligonucleotides, 1 μg of ivt mRNA having no (“A0”) or 120 (“A120”) As after the stop codon, 100 pmol of ECHO-53 per well in a total volume of 100 µL PBS and read at 480/520 nm. (**A**) shows the titration curve obtained for the A_60_ RNA oligo (“A60”). Individual fluorescence values for the A_60_ RNA oligo are plotted in blue, overlaid with the corresponding standard curve (pink solid line) and its 95% confidence interval (pink dotted lines). (**B**) are the raw fluorescence values obtained for the ivt mRNA samples, (**C**) shows the calculated amount of poly(A) in the ivt mRNA samples as calculated from the titration curve showed in (**A**) using interpolation by GraphPad Prism with a non-linear regression model, (**D**) shows the ratio of A stretches versus the mass of mRNA calculated by dividing the amount of poly(A) stretches in nanograms by the amount of mRNA in each well (1000 ng), and (**E**) shows the estimated poly(A) tail length (calculated by multiplying the length of the minimal mRNAs by the ratio shown in (**D**)).

To evaluate the length of poly(A) tails in synthetic mRNA, we used a range of 2–250 ng of Poly(A)_60_ RNA to generate a standard curve (Figure 2A), and in other wells, 1 μg of ivt mRNA coding gaussia luciferase, firefly luciferase or mCherry were used. These mRNAs were made from DNA templates having no (“A0”) or 120 (“A120”) in their transcribed region. As shown in Figure 2B, inclusion of the poly(A) sequence in the mRNA leads to increased fluorescence signal in the presence of ECHO-53. Using the established standard curve and interpolating the observed fluorescence values in a non-linear regression model using GraphPad Prism (for Windows, version 11.0.1, build 90) allows the calculation of the amount of contained poly(A) sequence in each well containing ivt mRNA. (Figure 2C). The percentage of A stretches was obtained by dividing the amount of poly(A) sequences shown in Figure 2C by the amount of ivt mRNA used in each well (Figure 2D). Multiplying this ratio by the length of the mRNA (by taking the length without poly(A) tail for simplifying the evaluation and accepting that this is an approximation: 569 nucleotides for Gluc, 723 nucleotides for mCherry and 1663 nucleotides for Fluc), we obtained an estimation of the average poly(A) tail length in each sample (Figure 2E). Supplementary Figure S3 describes the whole assay and calculations. The calculated average poly(A) tail lengths ranged from 60 to 100 bases. This is consistent with the expectation that not all mRNAs contain a full-length poly(A) sequence as encoded by the DNA template, likely reflecting premature disengagement of the RNA polymerase on homopolymeric stretches, particularly as ATP becomes limiting during transcription.

## 4. Discussion

The increasing prominence of ivt mRNA in vaccines and therapeutics necessitates the development of straightforward, cost-effective methods for quality control. One of the most critical quality attributes of synthetic mRNA is the length of its 3′ poly(A) tail, which is essential for ensuring mRNA stability and efficient translation. While traditional methods for measuring poly(A) tail length, such as reverse transcription and sequencing or capillary electrophoresis, are highly precise, they are also convoluted, labor-intensive, and not practical for many research laboratories. Our study introduces a simple, rapid, and economical alternative for estimating the average poly(A) tail length in ivt mRNA samples.

Our method is based on the distinctive properties of thiazole orange, a fluorescent dye that fluoresces intensely upon binding to nucleic acids [14,15,16,17]. We engineered an oligo(dT)_12_ deoxyoligonucleotide with thiazole orange molecules at both its 5′ and 3′ ends to create an ECHO probe (ECHO-53) that specifically hybridizes with the poly(A) tail of mRNA. The resulting fluorescence signal is directly proportional to the amount of poly(A) present in the sample. By generating a standard curve using known amounts of a poly(A)_60_ RNA oligonucleotide, we can easily and quickly translate the fluorescence signal from an mRNA sample into an estimated average poly(A) tail length. This approach provides a significant advantage over existing technologies, enabling researchers and small-scale production facilities to perform rapid quality control checks without the need for specialized equipment or extensive expertise.

The primary advantage of our method is its simplicity and speed. The entire assay can be completed in minutes using a standard microplate reader, making it ideal for high-throughput screening or routine quality control. This is a stark contrast to techniques like sequencing, which can take days to yield results. Furthermore, the method is highly accessible, relying on a simple hybridization reaction in a common laboratory buffer. The use of the double-labelled deoxyoligonucleotide (thiazole orange at both ends) was shown to significantly increase the signal-to-noise ratio, enhancing the sensitivity of the assay and allowing for reliable measurements with small amounts of mRNA.

Despite its clear advantages, our method provides an estimation rather than a precise measurement of poly(A) tail length. The calculation of the average tail length relies on several approximations, including the use of an mRNA length that excludes the poly(A) tail. This method evaluates the average length for the entire population of mRNA molecules in a sample and does not provide information on the distribution of poly(A) tail lengths, as is possible with sequencing. For instance, a sample with a mix of very short and very long tails might yield the same average as a sample with a more uniform distribution.

The need for a reliable and efficient quality control method is paramount in the rapidly expanding field of mRNA-based medicine. The inherent variability in the poly(A) tail length of ivt mRNA, whether from polymerase disengagement or post-transcriptional polyadenylation, makes routine quality assessment essential for ensuring product consistency and efficacy. Our method offers a practical solution for laboratories producing mRNA for research, pre-clinical studies, and even for personalized medicine applications where rapid, on-demand quality checks are crucial.

Future work could focus on further refining the assay to improve its precision and expand its utility. Validating the method against sequencing data for a broader range of mRNA constructs and poly(A) lengths would provide a more robust understanding of its accuracy. Moreover, exploring the use of different labelled deoxyoligonucleotide lengths or chemistries could potentially improve the assay’s dynamic range and sensitivity. Ultimately, our simple and efficient method fills a critical gap in the quality control workflow for synthetic mRNA production, providing a valuable tool for accelerating the development of next-generation mRNA therapeutics.

## Data Availability

Data associated with this paper can all be found within the article.

## Supplementary Materials

The following supporting information can be downloaded at https://www.mdpi.com/article/10.3390/mps9040109/s1: Figure S1: Effect of ionic strength; Figure S2: Effect of incubation time. Figure S3: Schematic showing how the poly(A) tail length calculations were determined.
