# Supplementary material for: A Simple Method to Evaluate the Length of Poly(A) Tails in mRNA
Source: Methods Protoc. 2026 Jul 13;9(4):109. doi: 10.3390/mps9040109 (PMC13398250; doi:10.3390/mps9040109)
Supplement: Supplementary file 1 [file mps-09-00109-s001.zip › mps-4389757-supplementary.pdf]

methods and protocols

MDPI

Technical Note

A Simple Method to Evaluate the Length of Poly(A) Tails in mRNA

Jonas Mumenthaler [1,2,†], Maximilian Feldmann [1,2,†], Shahab Mamaghani [1,3], Rocco Roberto Penna [1,3], Julia Frei [1,2], Natalia Jarzebska [1,3], Mark Mellett [1,2], Emmanuella Guenova [4], Thomas Kündig [1,2], Severin Lauchli [1,2,‡] and Steve Pascolo [1,2,*,‡]

[1] Department of Dermatology, University Hospital Zürich (USZ), University of Zürich (UZH), 8091 Zürich, Switzerland; jonas.mumenthaler@usz.ch (J.M.); maximilian.feldmann@usz.ch (M.F.); shahab.mamaghani@biomix.ch (S.M.); rocco.penna@usz.ch (R.R.P.); julia.frei@usz.ch (J.F.); mark.mellett@usz.ch (M.M.); thomas.kuendig@usz.ch (T.M.K.); severin.laeuchli@stadtspital.ch (S.L.); steve.pascolo@usz.ch
[2] Faculty of Medicine, University of Zürich, 8091 Zürich, Switzerland
[3] Faculty of Science, University of Zürich, 8091 Zürich, Switzerland
[4] Lausanne University Hospital (CHUV) and University of Lausanne, 3001 Lausanne, Switzerland; emmanuella.guenova@jku.at
* Correspondence: steve.pascolo@usz.ch
† These authors contributed equally to this work.
‡ These senior authors contributed equally to this work.

Academic Editor: Fernando Albericio

Received: 4 June 2026
Revised: 8 July 2026
Accepted: 10 July 2026
Published: 13 July 2026

Copyright: © 2026 by the authors. Submitted for possible open access publication under the terms and conditions of the Creative Commons Attribution (CC BY) license.

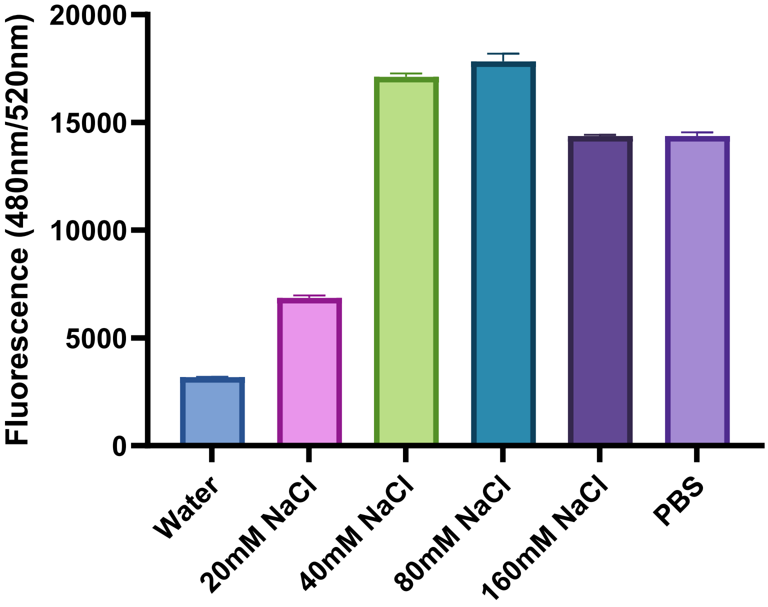

Figure S1. Effect of ionic strength.

The assay was performed in white 96 well plates with 500 ng of poly(A)$_{60}$ RNA oligonucleotides and 100 pmol of ECHO-53 deoxyoligonucleotide per well in a total volume of 100 µl of the indicated solutions and read at 480/520 nm.

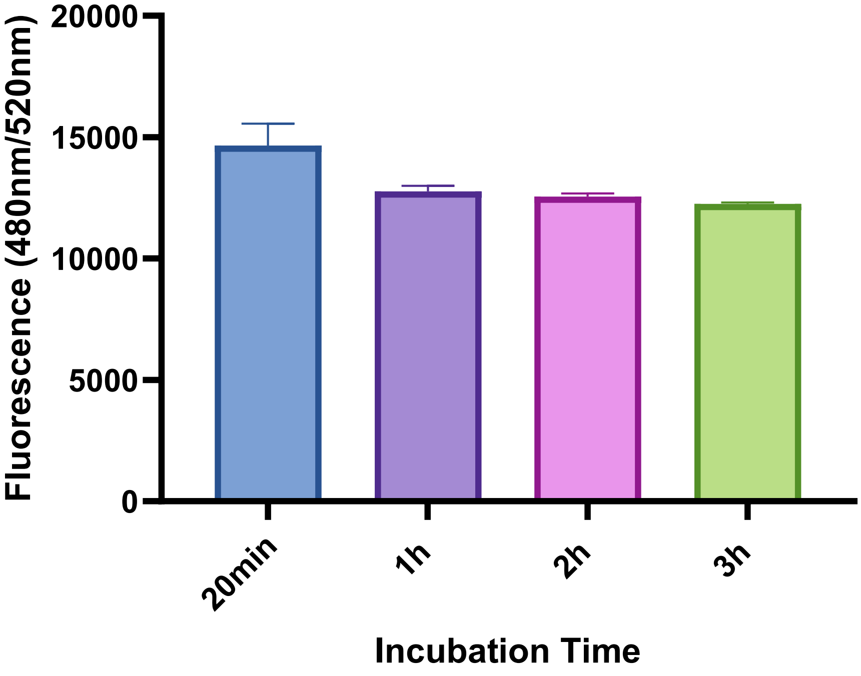

Figure S2. Effect of incubation time.

The assay was performed in white 96 well plates with 500 ng of poly(A)$_{60}$ RNA oligonucleotides and 100 pmol of ECHO-53 deoxy-oligonucleotide per well in a total volume of 100 µl of PBS and read at 480/520 nm at the indicated time post mixing.

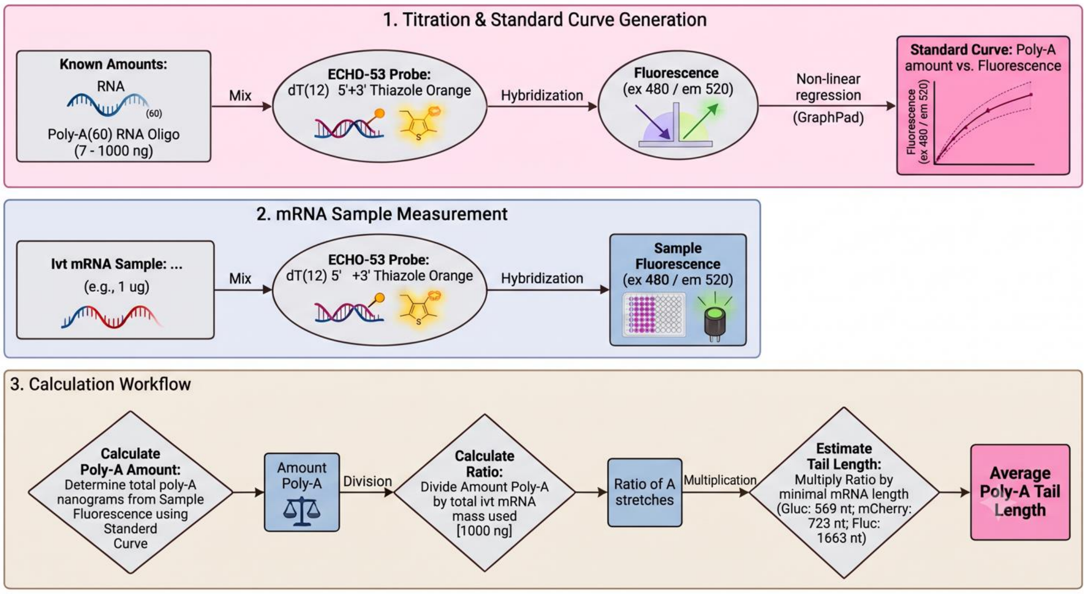

Figure S3. Schematic showing how the poly(A) tail length calculations were determined.
